# Pre-Harvest Sprouting Resistance in Bread Wheat: A Speed Breeding Approach to Assess Dormancy QTL in Backcross Lines

**DOI:** 10.3390/plants13233437

**Published:** 2024-12-07

**Authors:** Burak Ayık, Tuğba Güleç, Nevzat Aydın, Aras Türkoğlu, Jan Bocianowski

**Affiliations:** 1 Department of Bioengineering, Faculty of Engineering, Karamanoğlu Mehmetbey University, Karaman 70100, Türkiye; burakayik1@gmail.com; 2 Department of Plant and Animal Production, Vocational School of Technical Sciences, Karamanoğlu Mehmetbey University, Karaman 70100, Türkiye; tuba.eserkaya@gmail.com; 3 Department of Field Crops, Faculty of Agriculture, Necmettin Erbakan University, Konya 42310, Türkiye; 4 Department of Mathematical and Statistical Methods, Poznań University of Life Sciences, Wojska Polskiego 28, 60-637 Poznań, Poland

**Keywords:** wheat, dormancy, pre-harvest sprouting, speed breeding

## Abstract

In this study, BC1F3:4 generation plants derived from the hybrid crosses of Rio Blanco × Nevzatbey, Rio Blanco × Adana99, and Rio Blanco × line 127 were used as experimental material. These hybrids incorporated QTLs associated with pre-harvest sprouting (PHS) resistance through molecular techniques. Key agronomic traits, including plant height, spike length, the number of grains per spike, grain weight, and physiological maturity, were evaluated in both greenhouse and growth room settings under accelerated growth (speed breeding) conditions. Results indicated that plants grown in the fully controlled greenhouse conditions exhibited superior agronomic performance compared with those cultivated in the growth room. Additionally, germination tests were conducted on each hybrid cross to identify genotypes exhibiting dormancy. The analysis revealed that 11 lines from the Rio Blanco × Nevzatbey combination, eight lines from Rio Blanco × Adana99, and six lines from Rio Blanco × Line 127 had notably low germination indices. Among the three hybrid families, the Rio Blanco × Nevzatbey BC1F3:4 hybrids demonstrated the lowest germination index (0.44). Furthermore, Rio Blanco itself recorded the lowest germination index under both greenhouse (0.02) and growth room (0.24) conditions. These findings suggest that environmental conditions exert a significant influence on the expression of dormancy in these genotypes, even when dormancy genes are present. The lines developed in this research have the potential to serve as elite material in breeding programs aimed at enhancing pre-harvest sprouting resistance.

## 1. Introduction

Wheat (*Triticum aestivum* L.) is a critically important cereal crop that contributes substantially to global food security [1]. With a high nutrient content, wheat accounts for approximately 20% of daily caloric intake worldwide [2]. Wheat yield and quality are affected by numerous genetic and environmental factors, and for sustainable production, it is essential that the crop withstand various stressors [3]. Pre-harvest sprouting (PHS) of caryopses, particularly in coastal regions, is a notable factor impacting wheat quality. Climate change has intensified summer rainfall events, leading to higher PHS incidence not only in coastal but also in inland regions, causing severe yield losses [4]. Seeds that germinate within the spike before harvesting present substantial challenges in post-harvest processing [5].

The global annual economic loss due to PHS in wheat is estimated at roughly one billion dollars [6]. Consequently, developing genotypes with PHS tolerance has significant economic value. Breeding programs focused on producing genotypes with heightened seed dormancy, or even super dormancy, can play a critical role in mitigating PHS-related losses [7].

Pre-harvest sprouting (PHS) is influenced by seed dormancy, seed coat color, α-amylase activity, and plant growth hormones like abscisic acid, gibberellin, and auxin [8]. Seeds with dormancy exhibit reduced germination susceptibility under rainy harvest conditions [9]. Typically, while seeds have lower post-harvest dormancy than red-grain seeds, a trait linked to the pleiotropic effects of the *R-A1*, *R-B1*, and *R-D1* genes on chromosome 3, which impact the red color and influence dormancy [10]. However, some studies suggest that PHS tolerance can be independent of grain color [11,12].

Pre-harvest sprouting (PHS) is a complex quantitative trait with a significant genotype × environment interaction. The advancement of molecular techniques has led to significant progress in identifying genes involved in PHS and applying them in wheat breeding programs [13]. Approximately 29 QTLs associated with germination in pre-harvest spikes have been mapped across 11 chromosomes, excluding 1A, 2A, 7A, 5B, 6B, 1D, 4D, 5D, 6D, and 7D. These QTLs account for 23–78.3% of the observed phenotypic variation [13]. Kato et al. [14] developed a double haploid population by crossing a high dormancy variety with a low dormancy variety and identified three QTLs on chromosomes 4A, 4B, and 4D responsible for dormancy independent of grain color. These QTLs have become key candidates for PHS studies in breeding programs [12].

Rio Blanco is a white, hard grain, winter wheat renowned for its exceptionally high level of pre-harvest sprouting (PHS) tolerance [15]. A major QTL associated with this tolerance, identified as *QPhs.pseru-3AS*, was located on the short arm of chromosome 3A and was found to account for 41% of the phenotypic variation. Additionally, smaller QTLs on chromosome 2B were identified, contributing 5.0–6.4% of the phenotypic variation [16]. Breeding efforts to enhance pre-harvest sprouting (PHS) tolerance in white-grain wheat have shown promising results. In one study, QTL associated with high dormancy and independent of grain color was identified in white-grain wheat using molecular techniques. This QTL was named *TaMFT-3A* [17]. Shao et al. [18] conducted Genome-Wide Association Studies (GWAS) on the local white-grain variety Danby and identified the *Qphs.hwwg-5A.1* QTL, located on the long arm of chromosome 5A, as being associated with pre-harvest sprouting dormancy. Another study found that a QTL on chromosome 4A explained 40% of seed dormancy through mapping with close isogenic lines [19]. Additionally, Whole Genome Mapping in white-grain wheat revealed seven stable QTLs for germination resistance in pre-harvest spikes, located on chromosomes 1AS, 2AL, 2DL, 3AL, and 3BL [20]. In North American white winter wheat, pre-harvest sprouting resistance QTLs were identified on chromosomes 1AS, 3BL, 4AL, 5DL, and 6BL [21].

Due to the pleiotropic effect between the seed color and post-harvest dormancy, white kernel wheat is generally more susceptible to sprouting than red kernel wheat [22]. It is a significant advantage to prefer genotypes with red kernels and post-harvest seed dormancy in regions with high relative humidity. However, if some white-grained genotypes find a large cultivation area in risky areas, developing new varieties using white-grain dormant varieties becomes inevitable. Since the pre-harvest sprouting can adversely affect the agronomic characteristics of white-grained wheat and the milling and final product quality of the harvested grain. Breeders need effective and reliable methods to produce resistance genotypes to pre-harvest sprouting and measure pre-harvest sprouting. For the tests to measure the resistance to pre-harvest sprouting, there are the germination tests on the grain or the spike and the determination of the falling number. Germination tests can be performed in the laboratory for artificial climate conditions and in the field under the rain. As well as producing the resistance genotypes to pre-harvesting sprouting, the breeder can be used with speed breeding methods [23]. With red kernel wheat, it can be used as a genetic source for resistance to the preharvest sprouting in white kernel wheat. In plant breeding, shortening the generation time is crucial for saving time and labor, and research in this area has intensified in recent years. Using the speed breeding method, up to six generations per year have been achieved in bread wheat, durum wheat, rye, barley, and chickpea under standard greenhouse conditions [24]. The speed breeding method involves three main steps: extending the photoperiod, harvesting seeds before full maturity, and applying cold treatment to break seed dormancy early [25]. Genotypes developed through a speed breeding program can be tailored to specific target environments and populations, enabling the rapid production of lines before they are tested in the field for more complex traits such as yield. While field trials necessitate more time and resources, speed breeding provides a fast-track approach for phenotypic selection, thereby accelerating genetic gain [23]. However, it is crucial to evaluate the phenotypic traits of genotypes under greenhouse conditions and then confirm these traits under field conditions to ensure their reliability and effectiveness. This study aimed to evaluate the agronomic and germination traits of BC1F3:4 generations from the hybrid crosses of Rio Blanco × Nevzatbey, Rio Blanco × Adana99, and Rio Blanco × Line 127. These hybrids were developed using the speed breeding method and evaluated under controlled greenhouse conditions as well as in a growth room under speed breeding conditions.

## 2. Results

### 2.1. Data on Developmental Periods of Plants Grown Under Fully Controlled Greenhouse Conditions and in a Plant Growth Room

Developmental data were collected for the hybrid crosses of Rio Blanco × Line 127 BC1F3:4, Rio Blanco × Adana99 BC1F3:4, and Rio Blanco × Nevzatbey BC1F3:4, and their respective controls under both greenhouse and plant growth room conditions (Table 1 and Table 2).

In the greenhouse, day one was marked by the emergence of the first leaf across all plant groups. The third leaf appeared on day seven for the Rio Blanco × Nevzatbey and Rio Blanco plants, while other hybrids reached this stage by day eight. For stem elongation, the Rio Blanco and Rio Blanco × Adana99 genotypes exhibited the earliest growth, completing this stage in 25 days, while the Nevzatbey genotype had the latest stem elongation. The shortest period prior to heading was observed in the Rio Blanco × Adana99 hybrid (35 days), and the longest in Nevzatbey (56 days). The Rio Blanco × Adana99 and Rio Blanco × Nevzatbey hybrids had the shortest spike periods, while Nevzatbey had the longest. Among flowering times, the Rio Blanco × Adana99 hybrid was the earliest, whereas Kocabuğday and Nevzatbey were the latest. The Kırik parent had the latest harvesting period (119 days), while the earliest harvesting was observed in the Rio Blanco × Adana99, Rio Blanco × Nevzatbey, and Rio Blanco × Line 127 hybrids and the Rio Blanco and Adana99 genotypes (Table 1).

In the plant growth room, Rio Blanco showed the earliest third leaf emergence, with other genotypes reaching this stage in 7–8 days. The Rio Blanco × Adana99 hybrid had the shortest stem elongation, while the Nevzatbey genotype exhibited the latest elongation. The Rio Blanco × Adana99 hybrid completed the period prior to heading in the shortest time (33 days), and Nevzatbey in the longest (70 days). Among the genotypes, the Rio Blanco × Adana99 hybrid had the shortest spike and flowering times, whereas the Nevzatbey variety had the longest. Harvesting days for the genotypes in the plant growth room were recorded in the following order: Adana99, Rio Blanco, Rio Blanco × Adana99, Rio Blanco × Nevzatbey, Line 127, Rio Blanco × Line 127, Kırik, Nevzatbey, and Kocabğday (Table 2).

### 2.2. Agronomic Characteristics and Germination Index of Rio Blanco × Nevzatbey BC1F3:4, Rio Blanco × Adana99 BC1F3:4, and Rio Blanco × Line 127 BC1F3:4 Hybrids Grown in Fully Controlled Greenhouse Conditions and Plant Growth Room

Statistically significant differences at the 0.01 level were observed among the hybrid lines Rio Blanco × Nevzatbey BC1F3:4, Rio Blanco × Adana99 BC1F3:4, and Rio Blanco × Line 127 BC1F3:4 in traits such as plant height, spike length, the number of grains per spike, grain weight per spike, physiological maturity period, and germination index in both controlled greenhouse and growth room settings (Table 3, Table 4, Table 5, Table 6, Table 7 and Table 8).

In the fully controlled greenhouse, the Rio Blanco × Nevzatbey BC1F3:4 hybrid exhibited an average plant height of 63.8 cm, spike length of 6.8 cm, 22.1 grains per spike, 0.99 g of grain weight per spike, a physiological maturity period of 121.2 days, and a germination index of 0.48. The same genotype in the growth room had a plant height of 57.2 cm, spike length of 7.4 cm, 18.3 grains per spike, 0.60 g grain weight per spike, a physiological maturity period of 93.2 days, and a germination index of 0.44. In this hybrid, greenhouse conditions yielded greater plant height, grain weight, physiological maturity, and germination index, while spike length was greater in the growth room (Table 9, Table 10, Table 11, Table 12, Table 13 and Table 14).

For the Rio Blanco × Adana99 BC1F3:4 hybrid, the greenhouse environment resulted in a plant height of 56.6 cm, spike length of 6.6 cm, 21.8 grains per spike, 0.98 g grain weight per spike, 93.6 days to physiological maturity, and a germination index of 0.54. In the growth room, plant height was 50.2 cm, spike length 6.1 cm, grains per spike 17.9, grain weight per spike 0.56 g, physiological maturity period 79.5 days, and germination index 0.50. This hybrid also demonstrated increased plant height, spike length, grain weight, physiological maturity, and germination index in the greenhouse (Table 9, Table 10, Table 11, Table 12, Table 13 and Table 14).

The Rio Blanco × Line 127 BC1F3:4 hybrid had a plant height of 78.1 cm, spike length of 8.7 cm, 25.9 grains per spike, grain weight per spike of 1.15 g, 107 days to physiological maturity, and a germination index of 0.58 in the greenhouse. In the growth room, plant height was 66.8 cm, spike length 9.4 cm, grains per spike 26.1, grain weight per spike 0.79 g, physiological maturity period 96.1 days, and a germination index of 0.53. This hybrid showed higher plant height, grain weight per spike, physiological maturity, and germination index in the greenhouse, with greater spike length and grain number per spike in the growth room. Among the hybrids, Rio Blanco × Nevzatbey BC1F3:4 exhibited the lowest germination index (0.44) (Table 9, Table 10, Table 11, Table 12, Table 13 and Table 14).

### 2.3. Agronomic Characteristics and Germination Index of Control Varieties Grown Under Fully Controlled Greenhouse Conditions and Plant Growth Room

Among the control genotypes grown in the fully controlled greenhouse, the Rio Blanco variety displayed the shortest plant height (43.9 cm), while the Kırik variety was the tallest (121.8 cm). In the growth room, Rio Blanco remained the shortest at 48.6 cm, with Kırik again being the tallest (109.1 cm) (Table 9).

In terms of spike lengths, Rio Blanco had the shortest spike length in the greenhouse (6.4 cm), while the Karabuğday variety was the longest (10.4 cm). In the growth room, Adana99 had the shortest spike length (6.5 cm), while Kırik reached the longest (9.5 cm) (Table 10).

Regarding the number of grains per spike, the Line 127 variety had the lowest average number in the greenhouse, while the Kırik variety had the highest. In the plant growth room, the Rio Blanco variety had the minimum number of grains per spike, and Adana99 had the maximum (Table 11).

For grain weight per spike, the Nevzatbey variety had the lowest value in the greenhouse, whereas Kırik had the highest. In the plant growth room, Kırik again had the lowest average grain weight per spike, while Adana99 had the highest (Table 12).

The physiological maturation times in the greenhouse showed that the Rio Blanco variety had the shortest maturation period at 86.8 days, while Kırik had the longest at 119.4 days. In the plant growth room, Adana99 had the shortest maturation time, and Kırik had the longest (Table 13).

Germination index values in the greenhouse were as follows: Rio Blanco—0.02, Nevzatbey—0.16, Adana99—0.63, and Line 127—0.56. The Kocabuğday and Kırik genotypes, which are more sensitive to germination in pre-harvest sprouting, had higher values of 0.80 and 0.74, respectively. In the plant growth room, Rio Blanco had a germination index of 0.24, Nevzatbey 0.34, Adana99 0.56, Line 127 0.37, and Kocabuğday and Kırik had values of 0.72 and 0.43, respectively (Table 14). Overall, Rio Blanco had the lowest germination index in both environments.

## 3. Discussion

Analysis of the dataset revealed significant differences in plant height, physiological maturity, and various spike-related agronomic traits. In the Rio Blanco × Nevzatbey BC1F3:4 genotypes grown under greenhouse conditions, 52% of plants exhibited a germination index below 50, while this percentage increased to 66% in the plant growth room, indicating enhanced germination resistance in controlled growth room conditions. Among the Rio Blanco × Adana99 BC1F3:4 hybrids, 42% of plants showed a germination index below 50, with 48% in the growth room also exhibiting values under 50. For Rio Blanco × Line 127 BC1F3:4 lines, 28% of plants had a germination index below 50 in the greenhouse, compared with 45% in the plant growth room.

Despite the presence of germination-resistant QTLs in each hybrid group, the distribution of pre-harvest spike indices was approximately normal. The Rio Blanco × Line 127 BC1F3:4 and Rio Blanco × Adana99 BC1F3:4 hybrids exhibited higher germination rates, while the Rio Blanco × Nevzatbey BC1F3:4 hybrids showed lower germination rates. This normal distribution of pre-harvest sprouting index values likely results from significant genotype × environment interactions and low heritability for seed dormancy, posing challenges for the breeding of varieties resistant to pre-harvest sprouting.

This research is distinctive in its evaluation of pre-harvest spike germination resistance using speed breeding in controlled environments. Field testing of these lines will provide critical data to aid in selecting genotypes suited to specific target regions.

The study also included a comparative analysis of winter varieties such as Rio Blanco, Nevzatbey, and Line 127 with Adana99, a summer variety. The developed hybrids exhibited significant agronomic variation, including differences in plant height and heading time. These traits are fundamental for identifying genotypes with winter or summer growth characteristics, influencing their regional adaptability.

Tall and early-maturing genotypes are generally preferred in areas with low rainfall and no irrigation facilities. Their advantages include better drought escape mechanisms and increased carbohydrate storage in the stem during drought conditions [26]. Conversely, late-season and shorter-growing genotypes are favored in regions with ample rainfall and high yield potential due to their extended photosynthesis period and lodging resistance [27].

Research indicates that resistance to pre-harvest sprouting is often associated with grain color, with red grain genotypes typically showing greater resistance compared with white grain genotypes, which are more susceptible [11,12,22,28,29]. Yücel et al. [30] specifically reported challenges in preventing pre-harvest sprouting in white grain wheat.

This study, however, successfully addressed pre-harvest sprouting resistance in white-grained wheat, demonstrating that targeted strategies can effectively manage sprouting in genotypes traditionally considered more vulnerable. These findings underscore the potential to enhance sprouting resistance through breeding programs tailored for white-grain wheat.

The study further examined pre-harvest sprouting traits in three hybrid families with resistance QTLs introduced via molecular methods. These hybrids were phenotypically tested under speed breeding conditions in both fully controlled greenhouse and plant growth room settings.

Speed breeding technology has greatly accelerated the breeding cycle, allowing multiple annual generations, which is particularly beneficial for wheat breeding. Additionally, phenotypic testing in greenhouse conditions provides valuable insights into traits like plant height and heading time, which often exhibit comparable patterns in both greenhouse and field environments. This approach supports the efficient development and assessment of resistant genotypes, enhancing the overall breeding process.

## 4. Materials and Methods

### 4.1. Plant Material

The plant materials for this study were produced in a previous investigation [31]. The genotypes used were derived from backcross (BC) populations of the hybrid crosses: Rio Blanco × Nevzatbey, Rio Blanco × Adana99, and Rio Blanco × Line 127. The Rio Blanco variety is a white-grained wheat with strong resistance to pre-harvest sprouting and is classified as a winter type. The genetic basis for its resistance was identified through genotypic studies [16]. In contrast, the Nevzatbey and Line 127 varieties, both winter types, as well as the Adana99 variety, a summer type, do not exhibit tolerance to pre-harvest sprouting. However, these varieties are noted for their high protein quality and significant dormancy levels. Local varieties of Kırik and Kocabuğday, which are white-grained and classified as super-sensitive genotypes, typically have lower protein quality. To develop the BC1F2 populations, local varieties Nevzatbey, Line 127, and Adana99 were backcrossed with the XBarc321 QTL, which confers pre-harvest sprouting resistance and was introduced from the Rio Blanco variety. Molecular analysis confirmed the presence of the *XBarc321* QTL in the germination resistance gene region for the pre-harvest spike of each population [31]. For this study, BC1F3:4 genotypes derived from the Rio Blanco × Nevzatbey, Rio Blanco × Adana99, and Rio Blanco × Line 127 combinations (developed from the BC1F2 populations) were utilized. Germination tests included both parent varieties and the highly sensitive local controls Kocabuğday and Kirik (Table 15).

### 4.2. Conducting Trials in a Speed Breeding Room and Fully Controlled Greenhouse Conditions

Initially, seeds were stored at +4 °C for four days and then transferred to an incubator at +20 °C with 90–100% relative humidity for two days to promote germination. Following germination, seeds were returned to +4 °C for vernalization. The Rio Blanco × Adana99 and Rio Blanco × Line 127 seeds underwent 30 days of vernalization, while the Rio Blanco × Nevzatbey seeds were vernalized for 45 days. The Rio Blanco variety itself was vernalized for 45 days, while the Nevzatbey, Line 127, Kocabuğday, and Kırik varieties were vernalized for 30 days, and the Adana99 variety for 7 days. The controlled greenhouse environment was maintained at 28 °C with a relative humidity of approximately 60–70%. To extend the photosynthetic period, halogen lamps were set to operate from 17:00 to 02:00. A fan and heater system regulated the temperature as needed.

**Figure 1:**
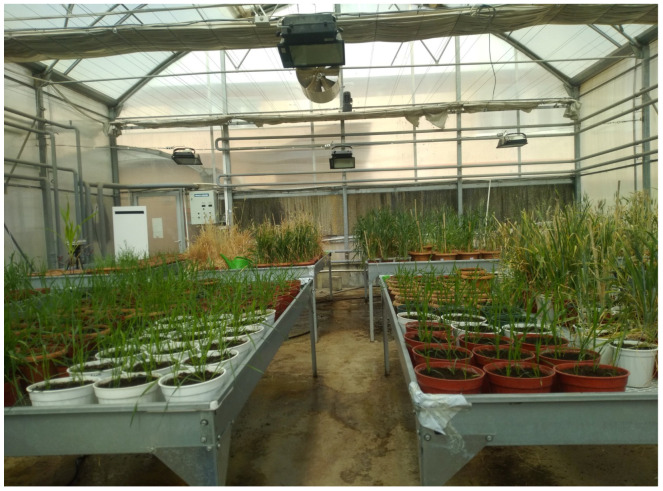
BC1F3:4 plants grown on tables under fully controlled greenhouse conditions; this figure illustrates the experimental setup, showcasing the arrangement of plants in the greenhouse to facilitate optimal growth conditions.

**Figure 2:**
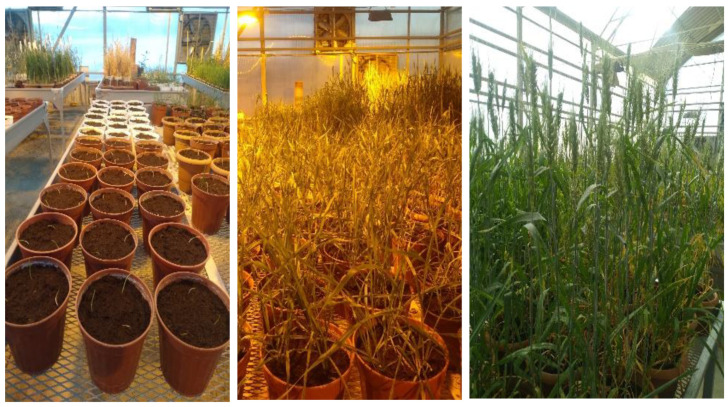
BC1F3:4 plants at different growth stages in fully controlled greenhouse conditions. This figure depicts the various developmental stages of the BC1F3:4 plants, showcasing their growth progress from emergence to maturity under optimal environmental conditions.

In the speed plant growth room, plants were exposed to 22 h of light and 2 h of darkness under LED lamps. The room temperature was maintained at 25 °C during the light period and 18 °C during the dark period, with relative humidity kept between 60–70%. Temperature stabilization was achieved using air conditioning, and humidity was controlled with a dehumidifier. Six tables, each measuring 90 × 180 cm, were arranged in the room (Figure 1). Two broad-spectrum LED lamps (160 W each) were placed on each table, with a 100 cm distance between the LEDs and the table surface (Figure 2).

Plants were irrigated daily with 200 mL of water for the first 20 days, followed by 500 mL thereafter. A combination fertilizer containing macro- and micronutrients was applied weekly at a rate of 1.5 g per 5 L, and foliar calcium nitrate was administered at 1 g per liter. Regular checks for aphids and powdery mildew were conducted, with necessary treatments applied as needed. Spikes harvested at physiological maturity were stored in a deep freezer at −20 °C to preserve dormancy. Observations were made on plant development stages, including first leaf emergence, third leaf emergence, stem elongation, pre-heading, heading, flowering, and harvest dates.

**Figure 3:**
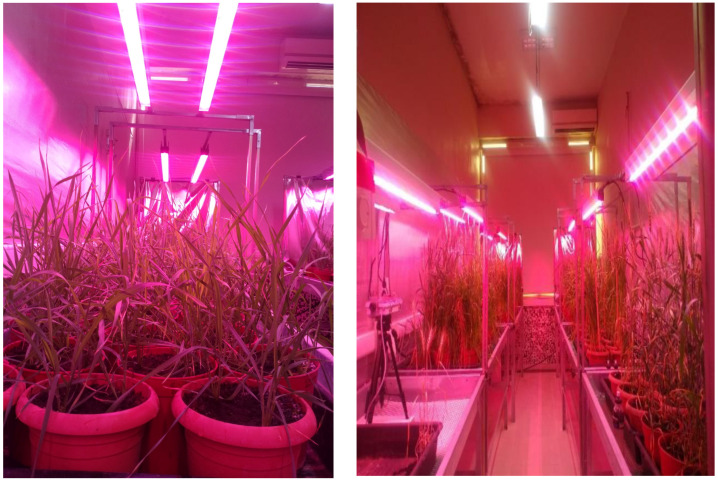
BC1F3:4 plants grown in the plant growth chamber. This figure illustrates the setup of the plant growth chamber, highlighting the conditions under which the BC1F3:4 plants were cultivated.

In the rapid growth chamber (Figure 3), a 22 h photoperiod was maintained using LED lighting, followed by a 2 h dark period. The temperature was controlled at 25 °C during the light period and 18 °C during the dark period. The LED lights were programmed to switch on at midnight (00:00) and remain on until 10:00 a.m., after which they were automatically turned off for a 2 h dark phase. During this time, the chamber was ventilated, allowing the plants to benefit from natural daylight. The lights were turned off again at 10:00 p.m. and resumed at midnight. The relative humidity in the rapid growth chamber was kept between 60–70%. Six tables, each measuring 90 × 180 cm, were arranged in the rapid breeding room for the study. Two 160 W broad-spectrum LED lamps were installed above each table, with a 100 cm gap between the LEDs and the table surface. An air conditioning unit was used to maintain a stable temperature in the room, and a dehumidifier was also installed to regulate humidity levels.

### 4.3. Determination of Physical Properties of Plant Materials

**Figure 4:**
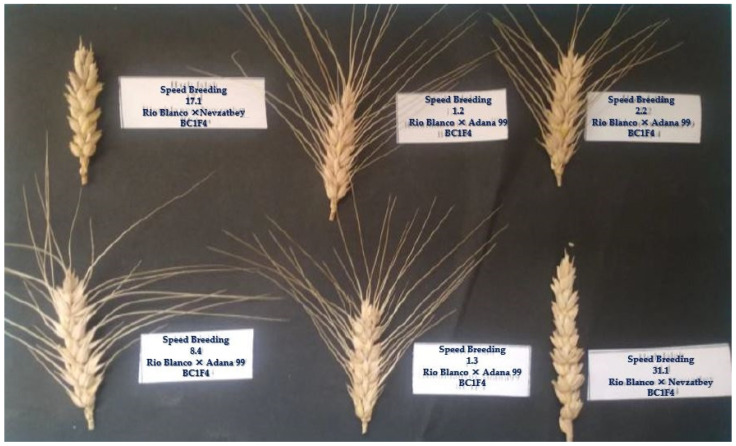
Spikes kept at room temperature prior to the germination test. This figure illustrates the preparation of the ears for testing, highlighting the importance of maintaining appropriate conditions before germination to ensure accurate test results.

Each parental, control, and backcross genotype were assigned a unique identifier, and individual plant tillers were numbered (Figure 4). Physiological maturity was assessed by observing the loss of green coloration in the spikes. Plant height and spike length were measured using a ruler.

### 4.4. Germination Analysis of Plant Materials

**Figure 5:**
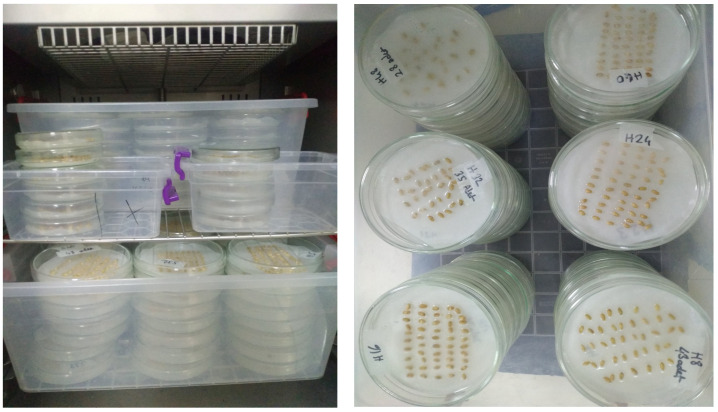
Seeds tested for germination in the incubator. This figure shows the setup of the incubator used for germination testing, illustrating the arrangement of seeds and any specific environmental conditions maintained, such as temperature and humidity.

To conduct germination tests, the spikes were threshed manually, and grain counts and weights were recorded. For each genotype, approximately 50 seeds were sterilized using 1% sodium hypochlorite and then rinsed with sterile water. Sterile Petri dishes lined with filter paper received 8–10 mL of sterile water for moisture consistency, and seeds were incubated in continuous darkness at 90–100% relative humidity. Over a seven-day period, germinated seeds were counted daily and removed (Figure 5). The germination index was calculated as follows:

$$Germination\;Index=\frac{7\times n1+6\times n2+5\times n3+4\times n4+3\times n5+2\times n6+1\times n7}{Total\;test\;days\times total\;number\;of\;grains}$$

where *n*_1_, *n*_2_, …, *n*_7_ represent the number of grains germinated on the first, second, … and seventh days of the germination test. The maximum germination index is 1.0, indicating that the genotype is not dormant, while the minimum value is 0.0.

**Figure 6:**
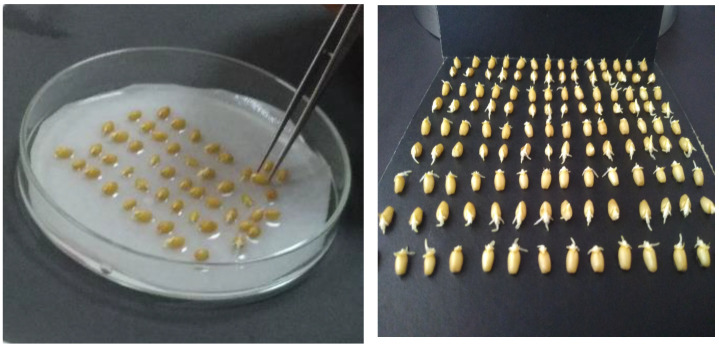
Counting the germinated seeds. This figure depicts the process of counting the seeds that have successfully germinated, highlighting the importance of accurate assessment in evaluating germination rates.

The germination test was conducted over a period of seven days. Germinated seeds were counted and removed from the Petri dishes (Figure 6). The germination index was then calculated using the formula provided below.

Genotypes germinating significantly within the first three days were classified as non-dormant and susceptible to pre-harvest sprouting [32].

### 4.5. Statistical Analysis

Plant height, spike height, number of grains, grain weight, and physiological maturity of the plants grown in the greenhouse and plant growth room, along with the data obtained from the germination test, were analyzed using the JMP statistical software v17.2 and Genstat 23.1 according to the augmented experimental design.

## 5. Conclusions

Agronomic data for the hybrid crosses were generally higher for plants cultivated under fully controlled greenhouse conditions compared with those in the plant growth room, likely due to the supplemental exposure to natural sunlight in the greenhouse environment. Among the genotypes examined, the Rio Blanco variety, used as a parent, exhibited the lowest germination index, while the Nevzatbey variety also displayed a lower germination index relative to other control varieties. In contrast, the local varieties Kocabuğday and Kırik, utilized as sensitive controls in the trials, consistently demonstrated higher germination index values. The results indicated that the speed breeding conditions exerted a considerable environmental effect on genotypes harboring QTLs linked to pre-harvest sprouting resistance. The lines identified with low germination indices in this study represent promising candidates for breeding programs aimed at enhancing resistance to pre-harvest sprouting, though further validation in field trials across varied environments is warranted. Additionally, these findings offer insights into the dormancy levels of the genotypes, aiding in the development of white wheat varieties with improved inherent germination characters.

## Figures and Tables

**Table 1:** Day data of the development periods of plants grown in the greenhouse.

Greenhouse									
Development Periods	Rio × Nb	Rio × Line 127	Rio × A99	Rio	Line 127	Nb	A99	Kırik	Kocabuğday
Planting date	18 February 2021								
1st leaf emergence	1	1	1	1	1	1	1	1	1
3st leaf emergence	7	8	8	7	8	8	8	8	8
Stem elongation	28	36	25	25	38	40	32	31	39
Prior to heading time	39	42	35	37	48	56	43	55	52
Heading time	37	44	37	42	50	59	46	58	54
Flowering time	46	52	40	44	54	60	51	59	60
Harvest date	106	107	93	93	110	115	94	119	113

Rio: Rio Blanco; Nb: Nevzatbey; A99: Adana99.

**Table 2:** Day data of the developmental periods of plants grown in the plant growth room.

Plant Growth Room									
Development Periods	Rio × Nb	Rio × Line 127	Rio × A99	Rio	Line 127	Nb	A99	Kırik	Kocabuğday
Planting date	18 February 2021								
1st leaf emergence	1	1	1	1	1	1	1	1	1
3st leaf emergence	7	8	8	5	8	8	8	8	8
Stem elongation	25	31	22	22	37	58	24	27	30
Prior to heading time	35	42	33	36	48	70	36	53	57
Heading Time	37	44	35	38	50	75	40	57	61
Flowering time	40	49	37	41	52	78	43	59	65
Harvest date	92	96	85	85	95	115	84	113	124

Rio: Rio Blanco; Nb: Nevzatbey; A99: Adana99.

**Table 3:** Analysis of variance tables regarding plant height (PH), spike length (SL), the number of grains per spike (NG), grain weight (GW), physiological maturity period (PMP), and germination index (GI) of the Rio Blanco × Nevzatbey hybrid grown in a fully controlled greenhouse.

Rio Blanco × Nevzatbey (Greenhouse)																		
Sources of Variation	^1^PH			SL			NG			GW			PMP			GI		
	SD	MS	F	SD	MS	F	SD	MS	F	SD	MS	F	SD	MS	F	SD	MS	F
Block	1	742.6	11.01	1	4.3	6.58	1	55.2	1.3	1	0.471	5.81	1	2.38	0.067	1	0.000075	0.652
Genotype	57	0.919	0.13 **	57	10.56	6.58 **	57	304.4	7.21 **	57	0.722	8.91 **	57	407.5	11.47 **	57	0.4347	3.78 **
Error	229	67.4		229	0.653		229	42.17		229	0.081		229	35.5		5	0.000115	
Total	287			287			287			287			287			63		

** 0.01 level of significance. ^1^PH: plant height. SL: spike length. NG: number of grains per spike. GW: grain weight. PMP: physiological maturity period. GI: germination index.

**Table 4:** Analysis of variance tables regarding plant height (PH), spike length (SL), the number of grains per spike (NG), grain weight (GW), physiological maturity period (PMP), and germination index (GI) of the Rio Blanco × Nevzatbey hybrid grown in a fully controlled plant growth room.

Rio Blanco × Nevzatbey (Plant Growth Room)																		
Sources of Variation	^1^PH			SL			NG			GW			PMP			GI		
	SD	MS	F	SD	MS	F	SD	MS	F	SD	MS	F	SD	MS	F	SD	MS	F
Block	1	43.1	0.907	1	0.313	0.383	1	42.1	0.923	1	0.145	1.959	1	11.99	0.456	1	0.00083	0.000005
Genotype	53	1041.7	21.91 **	53	7.86	9.60 **	53	171.1	3.75 **	53	0.273	3.68 **	53	816.6	31.09 **	53	0.0325	0.00021 **
Error	235	47.53		235	0.818		235	45.6		235	0.074		235	26.26		5	153.823	
Total	289			289			289			289			289			59		

** 0.01 level of significance. ^1^PH: plant height. SL: spike length. NG: number of grains per spike. GW: grain weight. PMP: physiological maturity period. GI: germination index.

**Table 5:** Analysis of variance tables regarding plant height (PH), spike length (SL), the number of grains per spike (NG), grain weight (GW), physiological maturity period (PMP) and germination index (GI) of the Rio Blanco × Adana99 hybrid grown in a fully controlled greenhouse.

Rio Blanco × Adana99 (Greenhouse)																		
Sources of Variation	^1^PH			SL			NG			GW			PMP			GI		
	SD	MS	F	SD	MS	F	SD	MS	F	SD	MS	F	SD	MS	F	SD	MS	F
Block	1	405.8	3.75	1	1.373	1.840	1	99.7	2.505	1	0.251	2.587	1	10.09	0.155	1	0.00007	0.067
Genotype	59	1611.3	14.902 **	59	9.45	12.667 **	59	144.4	3.628 **	59	0.41	4.226 **	59	484.9	7.494 **	59	0.00122	1.184 **
Error	210	108.1		210	0.746		210	39.8		210	0.097		210	64.7		5	0.00103	
Total	270			280			280			280			280			65		

** 0.01 level of significance. ^1^PH: plant height. SL: spike length. NG: number of grains per spike. GW: grain weight. PMP: physiological maturity period. GI: germination index.

**Table 6:** Analysis of variance tables regarding plant height (PH), spike length (SL), the number of grains per spike (NG), grain weight (GW), physiological maturity period (PMP), and germination index (GI) of the Rio Blanco × Adana99 hybrid grown in a fully controlled greenhouse and plant growth room.

Rio Blanco × Adana99 (Plant Growth Room)																		
Sources of Variation	^1^PH			SL			NG			GW			PMP			GI		
	SD	MS	F	SD	MS	F	SD	MS	F	SD	MS	F	SD	MS	F	SD	MS	F
Block	1	108.1	2.380	1	1.721	2.232	1	47.75	2.255	1	0.061	2.652	1	0.023	0.00049	1	0.00003	0.0121
Genotype	57	857.6	18.881 **	57	7.75	10.051 **	57	78.37	3.701 **	57	0.137	5.956 **	57	848.01	18.363 **	57	0.02823	11.429 **
Error	184	45.42		184	0.771		184	21.17		184	0.023		184	46.18		5	0.00247	
Total	242			242			242			242			242			63		

** 0.01 level of significance. ^1^PH: plant height. SL: spike length. NG: number of grains per spike. GW: grain weight. PMP: physiological maturity period. GI: germination index.

**Table 7:** Analysis of variance tables regarding plant height (PH), spike length (SL), the number of grains per spike (NG), grain weight (GW), physiological maturity period (PMP), and germination index (GI) of the Rio Blanco × Line 127 hybrid grown in a fully controlled greenhouse.

Rio Blanco × Line 127 (Greenhouse)																		
Sources of Variation	^1^PH			SL			NG			GW			PMP			GI		
	SD	MS	F	SD	MS	F	SD	MS	F	SD	MS	F	SD	MS	F	SD	MS	F
Block	1	0.036	0.00065	1	1.55	2.388	1	16.46	0.260	1	0.0243	0.192	1	23.13	0.569	1	0.0012	2.400
Genotype	57	1527	27.763 **	57	6.16	9.491 **	57	166.6	2.640 **	57	0.474	3.761 **	57	467.1	11.504 **	57	0.0433	86.600 **
Error	222	55		222	0.649		222	63.1		222	0.126		222	40.6		5	0.0005	
Total	280			280			280			280			280			63		

** 0.01 level of significance. ^1^PH: plant height. SL: spike length. NG: number of grains per spike. GW: grain weight. PMP: physiological maturity period. GI: germination index.

**Table 8:** Analysis of variance tables regarding plant height (PH), spike length (SL), the number of grains per spike (NG), grain weight (GW), physiological maturity period (PMP), and germination index (GI) of the Rio Blanco × Line 127 hybrid grown in a fully controlled plant growth room.

Rio Blanco × Line 127 (Plant Growth Room)																		
Sources of Variation	^1^PH			SL			NG			GW			PMP			GI		
	SD	MS	F	SD	MS	F	SD	MS	F	SD	MS	F	SD	MS	F	SD	MS	F
Block	1	78.6	1.738	1	0.679	0.786	1	14.69	0.430	1	0.0176	0.465	1	28.35	0.875	1	0.00013	0.00000037
Genotype	53	1178	26.06 **	53	0.197	0.228 **	53	263.3	7.707 **	53	0.2139	5.658 **	53	1282	39.580 **	53	0.03265	0.000093 **
Error	249	45.2		249	0.863		249	34.16		249	0.0378		249	32.39		249	349.8	
Total	303			303			303			303			303			303		

** 0.01 level of significance. ^1^PH: plant height. SL: spike length. NG: number of grains per spike. GW: grain weight. PMP: physiological maturity period. GI: germination index.

**Table 9:** Data on plant heights of genotypes grown in a fully controlled greenhouse and plant growth room.

Genotype	Plant Height (cm)					
	Greenhouse			Plant Growth Room		
	Min.	Max.	Mean	Min.	Max.	Mean
Rio × Nb BC1F3:4	37.0	100.2	63.8 ± 4.3	30.6	80.1	57.2 ± 3.4
Rio × A99 BC1F3:4	43.7	77.2	56.6 ± 5.8	38.2	66.8	50.2 ± 3.8
Rio × L.127 BC1F3:4	45.0	97.0	78.1 ± 3.9	42.4	84.3	66.8 ± 3.3
R.Blanco	43.9	49.2	46.0 ± 2.7	48.6	52.5	49.9 ± 2.3
Nevzatbey	78.9	81.1	80.2 ± 2.6	55.8	57.0	56.4 ± 2.4
Adana99	65.8	66.9	66.4 ± 2.8	47.3	55.1	51.2 ± 2.2
Line 127	57.2	63.8	59.9 ± 2.7	51.8	55.8	53.9 ± 2.2
Kocabuğday	101.9	109.7	105.6 ± 2.4	79.7	90.1	85.7 ± 2.0
Kırik	121.8	130.3	126.5 ± 2.5	98.4	109.1	104.3 ± 2.1

Rio: Rio Blanco; Nb: Nevzatbey; A99: Adana99; L.127: Line 127.

**Table 10:** Data on spike length of genotypes grown in a fully controlled greenhouse and plant growth room.

Genotype	Spike Length (cm)					
	Greenhouse			Plant Growth Room		
	Min.	Max.	Mean	Min.	Max.	Mean
Rio × Nb BC1F3:4	4.2	12.0	6.8 ± 0.42	4.4	10.5	7.4 ± 0.45
Rio × A99 BC1F3:4	5.0	9.8	6.6 ± 0.50	4.5	9.2	6.1 ± 0.49
Rio × L.127 BC1F3:4	7.2	10.8	8.7 ± 0.43	7.0	12.6	9.4 ± 0.40
R.Blanco	6.4	6.7	6.5 ± 0.30	7.0	7.3	7.2 ± 0.34
Nevzatbey	7.9	8.2	8.0 ± 0.30	7.4	8.2	7.5 ± 0.32
Adana99	7.7	8.4	8.0 ± 0.30	6.5	7.6	7.5 ± 0.30
Line 127	8.6	9.3	8.9 ± 0.30	8.4	9.2	8.6 ± 0.32
Kocabuğday	10.4	10.5	10.5 ± 0.20	8.4	9.4	9.4 ± 0.26
Kırik	10.1	10.7	10.4 ± 0.20	9.4	9.5	9.4 ± 0.29

Rio: Rio Blanco; Nb: Nevzatbey; A99: Adana99; L.127: Line 127.

**Table 11:** Data on the number of grains per spike of genotypes grown in a fully controlled greenhouse and plant growth room.

Genotype	Number of Grains per Spike (pieces)					
	Greenhouse			Plant Growth Room		
	Min.	Max.	Mean	Min.	Max.	Mean
Rio × Nb BC1F3:4	9.9	50.5	22.1 ± 3.5	1.3	34.0	18.3 ± 3.4
Rio × A99 BC1F3:4	13.8	37.4	21.8 ± 3.5	10.7	29.3	17.9 ± 2.6
Rio × L.127 BC1F3:4	12.8	39.1	25.9 ± 4.2	13.9	35.9	26.1 ± 2.8
R.Blanco	21.0	25.5	23.4 ± 2.2	10.2	12.1	11.4 ± 1.9
Nevzatbey	18.6	34.2	25.8 ± 2.1	18.7	20.5	19.7 ± 2.0
Adana99	24.8	26.2	25.5 ± 2.2	20.9	24.4	22.2 ± 1.9
Line 127	22.1	28.8	24.7 ± 2.2	15.7	19.7	17.9 ± 1.8
Kocabuğday	27.7	32.5	30.5 ± 1.9	15.6	18.0	16.7 ± 1.7
Kırik	30.8	32.7	31.9 ± 2.0	10.8	15.7	13.0 ± 1.8

Rio: Rio Blanco; Nb: Nevzatbey; A99: Adana99; L.127: Line 127.

**Table 12:** Data of grain weight per spike of genotypes grown in a fully controlled greenhouse and plant growth room.

Genotype	Grain Weight per Spike (g)					
	Greenhouse			Plant Growth Room		
	Min.	Max.	Mean	Min.	Max.	Mean
Rio × Nb BC1F3:4	0.38	2.35	0.99 ± 0.15	0.05	1.28	0.60 ± 0.14
Rio × A99 BC1F3:4	0.51	1.97	0.98 ± 0.17	0.26	1.15	0.56 ± 0.09
Rio × L.127 BC1F3:4	0.55	1.85	1.15 ± 0.19	0.31	1.34	0.79 ± 0.08
R.Blanco	1.06	1.36	1.23 ± 0.10	0.32	0.33	0.33 ± 0.07
Nevzatbey	0.45	1.68	1.04 ± 0.10	0.40	0.42	0.41 ± 0.07
Adana99	0.76	1.34	1.12 ± 0.10	0.59	0.81	0.73 ± 0.07
Line 127	0.84	1.33	1.10 ± 0.10	0.39	0.54	0.44 ± 0.06
Kocabuğday	1.08	1.62	1.33 ± 0.09	0.49	0.51	0.50 ± 0.06
Kırik	1.04	1.48	1.25 ± 0.09	0.21	0.44	0.32 ± 0.07

Rio: Rio Blanco; Nb: Nevzatbey; A99: Adana99; L.127: Line 127.

**Table 13:** Data on physiological maturity period of genotypes grown in a fully controlled greenhouse and plant growth room.

Genotype	Physiological Maturity Period (day)					
	Greenhouse			Plant Growth Room		
	Min.	Max.	Mean	Min.	Max.	Mean
Rio × Nb BC1F3:4	90.2	121.2	106.8 ± 3.1	70.1	117.7	93.2 ± 2.6
Rio × A99 BC1F3:4	86.4	107.8	93.6 ± 4.5	68.9	100.2	79.5 ± 3.8
Rio × L.127 BC1F3:4	93.4	129.6	107.0 ± 3.4	77.6	118.4	96.1 ± 2.76
R.Blanco	86.8	106.8	93.7 ± 2.1	48.6	52.5	84.5 ± 2.0
Nevzatbey	113.0	117.1	115.7 ± 2.1	55.8	57.0	114.0 ± 2.1
Adana99	88.8	95.0	94.5 ± 2.2	47.3	55.1	82.2 ± 1.9
Line 127	98.8	118.0	110.8 ± 2.1	51.8	55.8	94.2 ± 1.9
Kocabuğday	110.0	117.0	113.8 ± 1.9	79.7	90.1	124.8 ± 1.7
Kırik	119.4	120.6	119.9 ± 2.0	98.4	109.1	113.5 ± 1.8

Rio: Rio Blanco; Nb: Nevzatbey; A99: Adana99; L.127: Line 127.

**Table 14:** Data on germination index values of genotypes grown in a fully controlled greenhouse and plant growth room.

Genotype	Germination Index					
	Greenhouse			Plant Growth Room		
	Min.	Max.	Mean	Min.	Max.	Mean
Rio × Nb BC1F3:4	0.04	0.86	0.48 ± 0.010	0.11	0.72	0.44 ± 0.05
Rio × A99 BC1F3:4	0.12	0.86	0.54 ± 0.030	0.12	0.79	0.50 ± 0.05
Rio × L.127 BC1F3:4	0.02	0.81	0.58 ± 0.025	0.16	0.93	0.53 ± 0.01
R.Blanco	0.02	0.03	0.03 ± 0.016	0.24	0.29	0.27 ± 0.025
Nevzatbey	0.16	0.20	0.17 ± 0.016	0.34	0.37	0.35 ± 0.025
Adana99	0.63	0.65	0.64 ± 0.016	0.56	0.62	0.58 ± 0.025
Line 127	0.56	0.65	0.61 ± 0.016	0.37	0.55	0.45 ± 0.025
Kocabuğday	0.80	0.83	0.82 ± 0.016	0.72	0.73	0.72 ± 0.025
Kırik	0.74	0.78	0.76 ± 0.016	0.43	0.50	0.47 ± 0.025

Rio: Rio Blanco; Nb: Nevzatbey; A99: Adana99; L.127: Line 127.

**Table 15:** List of genotypes, responsible QTLs, and associated markers that control resistance to pre-harvest sprouting.

Name of Genotype	Generation	QTL-XBarc321
Rio × Nevzatbey	BC1F3:4	Exist
Rio × Adana99	BC1F3:4	Exist
Rio × L.127	BC1F3:4	Exist
R.Blanco	Genotype generator	Exist
Nevzatbey	Pure line parent	Nonexistent
Adana99	Pure line parent	Nonexistent
Line 127	Pure line parent	Nonexistent
Kocabuğday	Pure line parent	Nonexistent
Kırik	Pure line parent	Nonexistent

## Data Availability

All data supporting the conclusions of this article are included in this article.
